# Development and Characterization of Zein/Eudragit Composite Nanoparticles for Insulin Intranasal Delivery

**DOI:** 10.1021/acsomega.4c10474

**Published:** 2025-05-21

**Authors:** Felipe Figueiredo Moreira, Jeferson Ziebarth, Tatiane Patricia Babinski, Rubiana Mara Mainardes

**Affiliations:** † Laboratory of Nanostructured Formulations, 307046 Universidade Estadual do Centro-Oeste, Élio Antonio Dalla Vecchia Aveniu, 838, 85040-167 Guarapuava, Paraná, Brazil; ‡ Centro Universitário Campo Real, Comendador Norberto Street, 1299, 85015-420 Guarapuava, Paraná, Brazil; § Department of Pharmacy, Universidade Estadual do Centro-Oeste, Élio Antonio Dalla Vecchia Aveniu, 838, 85040-167 Guarapuava, Paraná, Brazil

## Abstract

Diabetes mellitus (DM) is a chronic metabolic disorder characterized by elevated blood glucose levels, primarily due to impaired insulin secretion or action. The standard treatment for Type 1 Diabetes Mellitus still involves daily parenteral insulin administration, which presents several challenges including patient discomfort, reduced adherence, and the potential for peripheral hyperinsulinemia. Intranasal administration has emerged as a promising alternative due to the nasal cavity’s high vascularization, ease of access, and significant absorption capacity, though certain physiological barriers remain. This study aimed to develop and characterize Zein-Eudragit nanoparticles (NPS) as carriers for insulin (ZEU/INS NPS) intended for intranasal administration. The NPS were prepared using a liquid–liquid dispersion method, and the production process was optimized through a 2^4^ factorial design. The resulting NPS were evaluated in terms of physicochemical properties, including particle size, polydispersity index (PDI), zeta potential, Fourier-transform infrared spectroscopy (FTIR), differential scanning calorimetry (DSC), thermogravimetric analysis (TG), and morphology. Additionally, the physical stability of the NPS during storage, in vitro insulin release, and in vitro mucoadhesion were assessed. The optimized nanoparticle formulation exhibited a mean particle size below 200 nm, a PDI of less than 0.3, a zeta potential of approximately +30 mV, and an encapsulation efficiency of 42%. FTIR analysis confirmed the interaction between nanoparticle components following insulin encapsulation, and DSC/TG analysis demonstrated the thermal stability of the system. NPS stored under refrigerated conditions maintained their stability for up to 60 days. In vitro release studies revealed that about 60% of the encapsulated insulin was released over a 24 h period. The in vitro mucoadhesion assay further supported the potential of these NPS to enhance the residence time in the nasal cavity. Overall, ZEU/INS NPS successfully demonstrated favorable physicochemical characteristics for the intranasal delivery of insulin. These findings suggest that these NPS offer significant promise as an effective and noninvasive delivery system for insulin.

## Graphical abstract

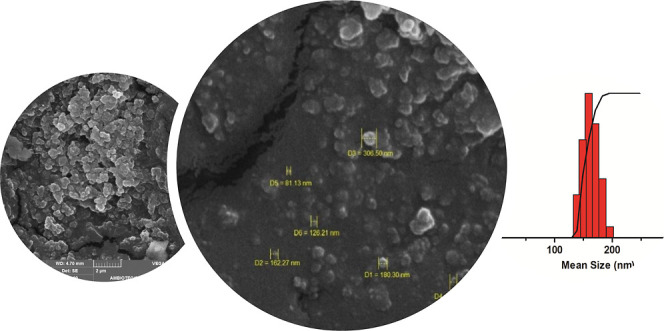

## 1 Introduction

The daily administration of insulin remains the primary therapy for patients with Type 1 Diabetes Mellitus (T1DM).^1^ Parenteral injection continues to be the most commonly employed route of insulin delivery, despite its associated limitations, including patient discomfort due to needle use, reduced treatment adherence, and peripheral hyperinsulinemia, which can exacerbate insulin resistance in muscle and peripheral tissues.^2,3^ These challenges underscore the pressing need for alternative administration routes that enhance patient compliance, ensure systemic insulin delivery, and improve bioavailability.

Intranasal delivery has emerged as a promising alternative for insulin administration, offering advantages such as rapid absorption, ease of access to the nasal cavity, and the potential for noninvasive application. The highly vascularized nasal mucosa facilitates effective drug absorption while bypassing first-pass hepatic and intestinal metabolism. Despite these advantages, intranasal insulin delivery faces hurdles such as mucociliary clearance, enzymatic degradation, and limited permeability to large biomolecules, which must be overcome to ensure effective delivery.^4,5^

In recent years, significant efforts have been made to enhance insulin absorption through the nasal route, with the application of nanoparticles (NPS) as carriers showing substantial promise.^6,7^ The field of nanotechnology has seen remarkable growth,^8,9^ particularly in nanomedicine, where NPS offer distinct advantages in drug delivery due to their ability to traverse microcapillaries and epithelial barriers, improve drug stability, and penetrate mucosal barriers more efficiently.^10,11^

Among the many nanoparticle systems explored, plant-protein-based NPS offer an innovative, biocompatible, and cost-effective solution for drug delivery. These proteins provide abundant functional groups, enabling surface modifications that optimize drug encapsulation and release.^12^

Zein, a hydrophobic plant protein derived from corn (Zea mays L.), is particularly notable for its ability to form NPS, making it an attractive vehicle for the controlled delivery of biomolecules such as insulin.^10,11^ Furthermore, zein NPS can encapsulate hydrophilic, hydrophobic, and amphiphilic compounds without compromising their bioactivity, offering flexibility for a variety of therapeutic applications.^13^ Despite these advantages, zein tends to aggregate due to hydrophobic interactions and lacks a sufficient positive surface charge for robust mucoadhesion.^14,15^ To overcome these limitations, Eudragit RS100, a cationic polymer, was incorporated into the formulation.

Eudragit RS100 provides a strong positive surface charge, enabling enhanced electrostatic interactions with the negatively charged mucin in the nasal cavity.^16^ This improves mucoadhesion, prolongs the residence time, and facilitates drug absorption. Furthermore, Eudragit RS100 stabilizes the NPS, reducing aggregation and contributing to a controlled drug release profile due to its polymeric matrix.^17^

In this study, we aim to develop and characterize Zein/Eudragit RS 100 composite NPS as an innovative delivery system for insulin, focusing on their potential for intranasal administration. This approach seeks to address the current limitations of parenteral insulin therapy by providing a noninvasive, efficient, and patient-friendly alternative.

## 2 Materials and Methods

### 2.1 Materials

Zein (19–22 kDa) and Mucin Type II were obtained from Sigma-Aldrich (Brazil), Eudragit RS 100 (150–300 kDa) was acquired from Evonik Industries (Germany), and insulin was provided by Novolin R human insulin, purchased from a commercial pharmacy in Guarapuava/PR—Brazil. All other reagents and chemicals were of analytical grade for HPLC. Water was purified using a Milli-Q Reagent grade water system (Millipore Corporation, Bedford, MA).

### 2.2 Preparation of Zein and Eudragit Nanoparticles Containing Insulin (ZEU/INS NPS)

The formation of ZEU/INS NPS follows a modified liquid–liquid precipitation method,^18^ which relies on the interplay of solubility changes, self-assembly, and interfacial interactions during solvent exchange.

Initially, zein (20 mg/mL) and insulin (700 μg/mL) were dissolved in an organic solvent system (85% ethanol) to form a homogeneous solution and incubated in a shaker at 150 rpm and 25 °C for 2 h. Subsequently, Eudragit RS 100 (200 mg/mL) was dissolved in the same organic solvent, added to the zein-insulin mixture, and maintained stirring at 150 rpm for 30 min. After stirring, this phase was slowly poured into 6 mL of ultrapure water under vigorous magnetic stirring (1500 rpm) for 30 min, inducing the precipitation of the components, driven by their decreased solubility in water compared to ethanol. The resulting nanoparticle suspension was subjected to complete ethanol removal by rotary evaporation at 50 °C. The final ZEU/INS NPS suspension was centrifuged at 15,000 rpm for 25 min at 25 °C. The supernatant was collected to assess encapsulation efficiency (EE), while the nanoparticle precipitate was resuspended in ultrapure water.

Blank NPS (ZEU NPS) were prepared following the same protocol, omitting insulin from the organic phase.

### 2.3 Optimization of the ZEU/INS NPS Formulation

#### 2.3.1 Factorial Design

To optimize the formulation parameters and achieve the ideal proportions between the components, a factorial design approach was employed, following the methodology described by Dey.^19^ A 2^4^ factorial design with one central point was utilized to investigate the effects of four independent variables on the nanoparticle characteristics.

The design involved 16 experimental runs conducted in triplicate, along with 5 additional runs at the central point to estimate pure experimental error, resulting in a total of 22 experiments. The independent variables studied were zein concentration (X1), Eudragit concentration (X2), organic-to-aqueous phase ratio (O/A) (X3), and incubation time (X4), each tested at two levels (−1 and +1), with one central point (0). The dependent variables were the mean particle size (R1), polydispersity index (PDI) (R2), zeta potential (R3), and EE (R4). Table 1 presents the independent and dependent variables used in the factorial design.

**Table 1:** Factors and Levels Used in the 2^4^ Factorial Design Experiment to Obtain the ZEU/INS NPS for α = 95%

		levels		
factor	independent variables	–1	0	+1
X1	zein concentration (mg/mL)	15	20	25
X2	Eudragit concentration (mg/mL)	15	20	25
X3	O/A ratio (v/v)	1/2	1/3	1/4
X4	incubation time (h)	1	2	3

	dependent variables
R1	average diameter (nm)
R2	polydispersity index
R3	zeta potential (mV)
R4	encapsulation efficiency (%)

### 2.4 Physicochemical Characterization

#### 2.4.1 Mean Particle Size and Zeta Potential

The mean particle size and PDI of the NPS were determined by using photon correlation spectroscopy, also known as Dynamic Light Scattering. A BIC 90 Plus instrument (Brookhaven Instruments Corp.) was employed for these measurements, providing an assessment of the hydrodynamic radius of the particles in the suspension. Measurements were conducted at a scattering angle of 90° at 25 °C using a laser with a wavelength of 660 nm.

The zeta potential was analyzed using a ZetaSizer ZS instrument (Malvern), operated at 25 °C with a potential of ±150 mV to determine the surface charge of the NPS.

#### 2.4.2 Determination of Insulin Encapsulation Efficiency

Insulin EE was assessed using an indirect method by quantifying the insulin remaining in the supernatant post centrifugation of the nanoparticle suspension. This was done using High-Performance Liquid Chromatography coupled with a Diode Array Detector (HPLC-DAD) (Waters Alliance), under established chromatographic conditions. The mobile phase consisted of acidified water (0.5%) and acetonitrile (60:40, v/v) with a C18 column (Xterra Waters, 250 × 4.6 mm, 5 μm). The flow rate was set to 1.0 mL/min, with an injection volume of 20 μL, a column temperature of 30 °C, and detection at 271 nm. The method was validated in-house to ensure accuracy. EE was calculated using eq 1

1 $$EE\;\left(\%\right)=\frac{IC-SC}{IC}\times 100$$

where IC represents the initial concentration of insulin and SC is the concentration of insulin in the supernatant.

#### 2.4.3 Scanning Electron Microscopy (SEM)

The morphology of the NPS was evaluated by using Scanning Electron Microscopy (SEM) with a Vega 3 microscope (Tescan) at an acceleration voltage of 20 kV. Samples were prepared by depositing a drop of nanoparticle dispersion on a metallic stub, followed by solvent evaporation. The dried samples were then coated with a thin layer of gold under a vacuum to improve electrical conductivity. SEM images were obtained at magnifications of 15,000 and 30,000×, allowing for detailed observation of the particle morphology, size distribution, and agglomeration patterns.

#### 2.4.4 Fourier Transform Infrared (FTIR) Spectroscopy

FTIR was used to assess the chemical composition of the NPS and their components, including insulin, zein, and Eudragit and a physical mixture of zein and Eudragit in a 1:1 ratio. The lyophilized powders of ZEU NPS and ZEU/INS NPS were also analyzed. Infrared spectra were recorded in the range of 650–4000 cm^–1^ using the Attenuated Total Reflectance method, which allowed for direct analysis of the samples without extensive preparation. The FTIR analysis was performed by using a PerkinElmer spectrometer to detect characteristic absorption bands, confirming chemical interactions and the integrity of the encapsulated components.

#### 2.4.5 Thermal Analyses (TG/DSC)

TG and DSC were conducted to assess the thermal stability and behavior of the NPS and their components. TGA was performed with a TGA 4000 instrument (PerkinElmer) under a nitrogen atmosphere at a flow rate of 100 mL/min. Approximately 4 mg of each sample (zein, Eudragit, physical mixture, ZEU NPS, and ZEU/INS NPS) was heated from 30 to 800 °C at a rate of 10 °C/min. DSC analysis was carried out using a DSC 4000 (PerkinElmer) from 30 to 400 °C, under the same atmospheric conditions. The Pyris software was used for data analysis.

### 2.5 Evaluation of Nanoparticle Stability

Nanoparticle stability was monitored over two months under different storage conditions: room temperature and refrigeration (10 °C). Three distinct nanoparticle suspensions were evaluated weekly for particle size, PDI, and zeta potential. Additionally, the insulin release profile was assessed monthly by measuring the supernatant after ultracentrifugation using HPLC.

### 2.6 In Vitro Release

In vitro release study was conducted in triplicate using a Franz diffusion cell system. Simulated nasal fluid was prepared with sodium chloride (NaCl), potassium chloride (KCl), and calcium chloride (CaCl_2_) at pH 6.5.^20,21^ The receptor chamber was filled with 7 mL of the simulated nasal fluid and maintained at 37 °C with constant agitation at 300 rpm. Approximately 95 μg of insulin-loaded NPS was applied to a 0.45 μm nitrocellulose membrane, and 1 mL samples were collected from the receptor medium at predefined intervals (30 min, 1 h, 2 h, 4 h, 8 h, 12 h, 24 h). Each sample was filtered using a 0.22 μm membrane and analyzed by HPLC. Release kinetics were analyzed using KinetDS software, applying various mathematical models, including Zero-order, First-order, Higuchi, Weibull, and Korsmeyer-Peppas models, to determine the release mechanism.^22^

### 2.7 In Vitro Mucoadhesion Study

To evaluate mucoadhesion, mucin solutions were prepared at concentrations of 100, 250, and 500 μL mL^–1^ in a 0.02 mol·L^–1^ phosphate buffer at pH 6.8. NPS (20 μL) were added to 1 mL of mucin solution and incubated at 37 °C in a shaker at 70 rpm for 30 min. Mean particle size, zeta potential, and PDI were measured before and after exposure to the mucin solutions.

### 2.8 Statistical Analysis

All data were expressed as mean ± standard deviation. Statistical significance was determined using one-way Analysis of Variance (ANOVA) followed by Tukey’s post hoc test for multiple comparisons. Statistical analysis was performed using Minitab software (Minitab Software, USA), with *p*-values <0.05 considered statistically significant.

## 3 Results and Discussion

### 3.1 Optimization of ZEU/INS NPS Formulation Using a Factorial Design Approach

A total of 22 experiments were performed to evaluate the influence of the process variables on the response variables: Mean Particle Size (nm) (R1), PDI (R2), Zeta Potential (mV) (R3), and EE (%) (R4). The detailed experimental results are shown in Table 2.

**Table 2:** Results of the Dependent Variables and Global Response (GR) for the Optimization of the ZEU/INS NPS Formulation

experiment	mean size (nm)	polydispersity index	zeta potential (mV)	encapsulation efficiency (%)	GR
1	383	0.34	+20	30	2.01
2	278	0.25	+18	4	1.14
3	1605	0.31	+25	12	2.34
4	568	0.29	+21	20	1.84
5	288	0.53	+18	27	2.12
6	677	0.2	+32	24	2.15
7	1798	0.56	+22	6	2.62
8	603	0.35	+19	12	1.7
9	342	0.35	+19	22	1.78
10	351	0.3	+20	15	1.58
11	267	0.41	+22	1	1.42
12	191	0.25	+35	20	1.96
13	418	0.44	+20	32	2.23
14	331	0.25	+29	23	1.94
15	1479	0.65	+24	9	2.7
16	321	0.27	+23	26	1.86
17	103	0.3	+31	39	2.39
18	142	0.28	+28	40	2.25
19	122	0.31	+30	35	2.21
20	184	0.23	+29	40	2.23
21	133	0.28	+30	39	2.27
22	115	0.28	+28	42	2.26

The mean particle size ranged from 103 to 1798 nm, highlighting significant variability across the experiments. Particles with sizes of ≤200 nm, observed in experiments 17, 18, 19, 20, 21, and 22, are particularly suitable for intranasal applications due to their enhanced ability to traverse biological barriers. Conversely, particles exceeding 1000 nm, as seen in experiments 3, 7, and 15, may reflect suboptimal interactions among the formulation components or aggregation during nanoparticle formation. Variations in zein concentration were found to significantly influence particle size; higher concentrations of zein led to larger particles, likely due to increased protein–protein interactions and aggregation during the precipitation process.^23,24^ This increase in size was often accompanied by higher PDI values, indicating a reduced uniformity in particle size distribution. The PDI, a measure of size distribution uniformity, ranged from 0.20 to 0.65. Lower PDI values (≤0.3), observed in several experiments, indicated homogeneously distributed particles, which are critical for achieving stability and consistent pharmacokinetic behavior. In contrast, PDI values exceeding 0.5, such as those seen in experiments 5, 7, and 15, suggested significant heterogeneity that could compromise reproducibility and stability. This heterogeneity is often linked to higher concentrations of zein or imbalanced phase ratios during nanoparticle preparation.

**Table 3:** Response Optimization

experiment	zein (mg)	Eudragit (mg)	O/A ratio (v/v)	incubation time (h)	EE (%) adjustment	potential zeta (mV) adjustment	PDI adjustment	mean size adjustment	composite desirability
1	20	20	1/3	2	40.3226	30.2989	0.299964	115.234	0.916447

The zeta potential ranged from +18 to +35 mV, indicating a consistently positive surface charge on the NPS. Zeta potentials above +25 mV, as observed in experiments 6, 12, 14, and 17–22, are highly desirable as they confer enhanced colloidal stability by promoting electrostatic repulsion and preventing particle aggregation. The surface charge was strongly influenced by the concentration of Eudragit RS100, which contains quaternary ammonium groups responsible for the positive charge.^25,26^ Optimal concentrations of Eudragit RS100 improved the zeta potential while also aiding in the stabilization of NPS. Excessive amounts, however, could lead to aggregation and higher PDI values.

The ratio of organic to aqueous phases (O/A) significantly affected the particle size and PDI due to its role in solvent diffusion and precipitation dynamics. Lower O/A ratios, where the aqueous phase is predominant, facilitate rapid solvent exchange and nanoparticle precipitation, resulting in smaller and more uniform particles with reduced PDI. In contrast, higher O/A ratios, dominated by the organic phase, slowed the diffusion process, leading to larger particles with higher PDI values. Despite these variations in size and uniformity, the zeta potential remained relatively stable, as it was more directly influenced by Eudragit’s contribution to surface charge rather than the phase ratio.

Incubation time was another critical factor influencing nanoparticle characteristics. Prolonged mixing times between zein, Eudragit RS100, and insulin improved particle uniformity and reduced PDI by enhancing component interaction. However, excessive incubation times (e.g., >3 h) led to particle aggregation and increased particle size, likely due to prolonged exposure to stirring and potential destabilization of the formulation. Notably, zeta potential remained largely unaffected by incubation time, provided that the polymer concentrations were consistent throughout the process.

To achieve simultaneous optimization of the ZEU/INS NPS formulation, the individual responses were combined into a GR. This GR was calculated by normalizing each response relative to the highest observed value, followed by summing all of the normalized values. The detailed experimental results are shown in Table 2.

Following completion of the experimental runs, a mathematical model was fitted to the data using GR as the dependent variable. The model was generated using Minitab software, applying multiple regression analysis, ANOVA, and calculation of *F*-values (Fisher distribution) and *p*-values. These statistical tools were employed to assess the appropriateness of the model in describing the experimental data, enabling the evaluation of the regression’s significance and the quality of the fit to the response variables.

The ANOVA results indicated that the linear model used was suitable for representing the experimental data, as evidenced by the absence of a significant lack of fit (*p*-value >0.05, with *p* = 0.1 > 0.05). Furthermore, the calculated F-value for the regression (1.31) was lower than the tabulated *F*-value (2.61), confirming the model’s adequacy.

The robustness of the model was further supported by the high coefficient of determination (*R* ^2^) obtained, which reached 98.07%. This value indicates that the model explains a substantial portion of the variance in the response data, suggesting that it is an effective tool for interpreting the experimental results. Based on these findings, it can be concluded that the linear model provides a reliable description of the experimental data.

#### 3.1.1 Regression Equation for Global Response (GR)

The regression equation derived from the factorial design analysis for the GR is as follows

globalresponse=1.880−0.0230zein+0.0179eudragit+0.1821O/Wratio−0.0343incubationtime+0.201Ct

To assess the quality of the linear model fit, residual plots were generated (Figure 1). The normal probability plot (A) reveals that the residuals closely align with a normal distribution, indicating conformity to the assumption of normality. The adjusted versus fitted values plot (B) demonstrates a random dispersion of residuals around zero, with no evident patterns or heteroscedasticity, underscoring the model’s validity. The histogram (C) further corroborates the normality assumption by displaying a symmetrical distribution centered at zero. Moreover, the residuals versus order plot (D) substantiates the independence of residuals, as no discernible patterns or trends are observed across sequential data points. Collectively, these diagnostic evaluations confirm that the residuals are well-standardized, providing strong evidence of the adequacy and robustness of the linear model for the data under analysis.

**Figure 1:**
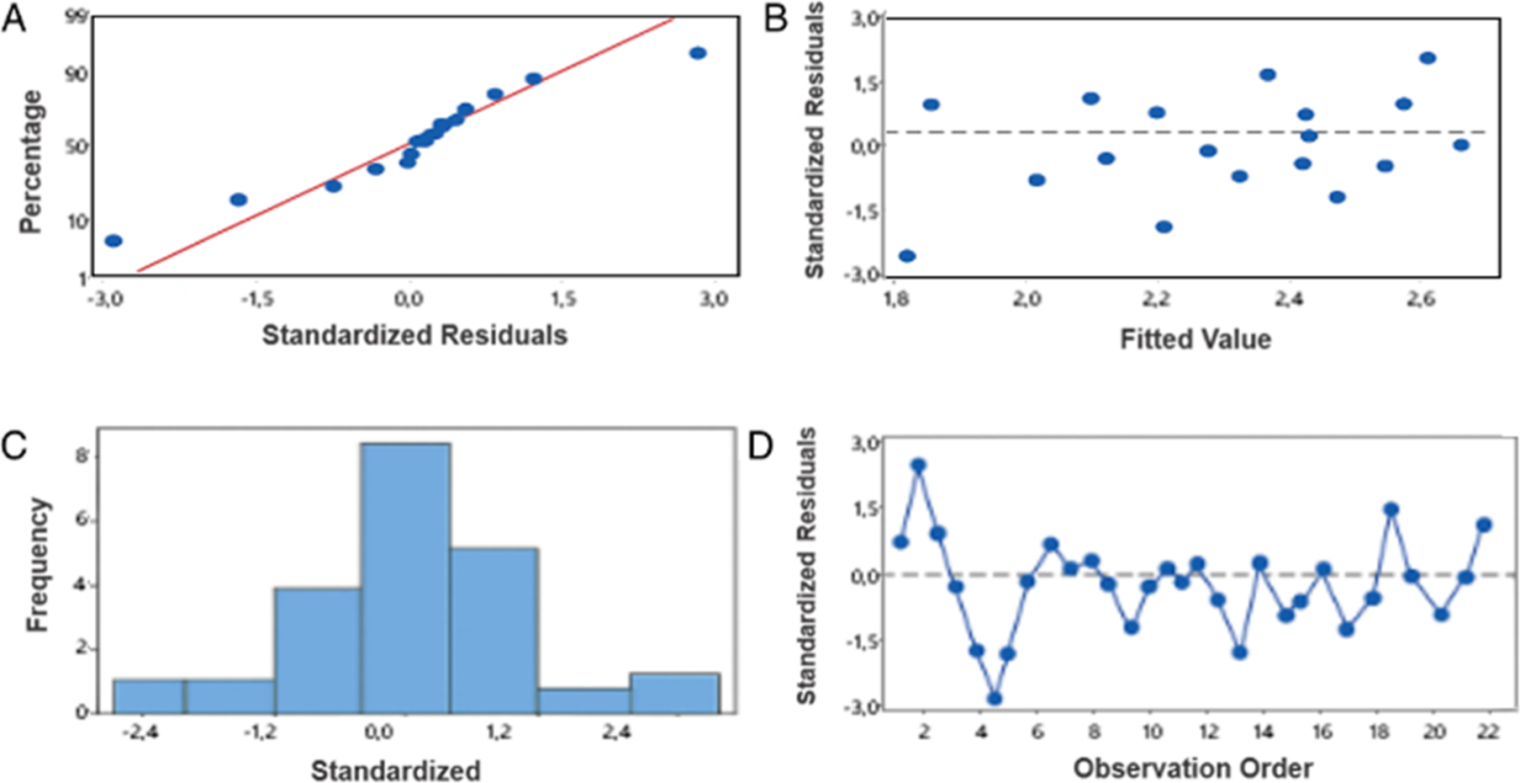
GR Residual Plot. (A) Probability plot, (B) adjusted versus fitted values plot, (C) histogram, (D) residuals versus order plot.

The design analysis allowed for the identification of significant factors that influence the GR. Using the Pareto chart (Figure 2), we identified the most relevant factors and interactions affecting the nanoparticle preparation process.

**Figure 2:**
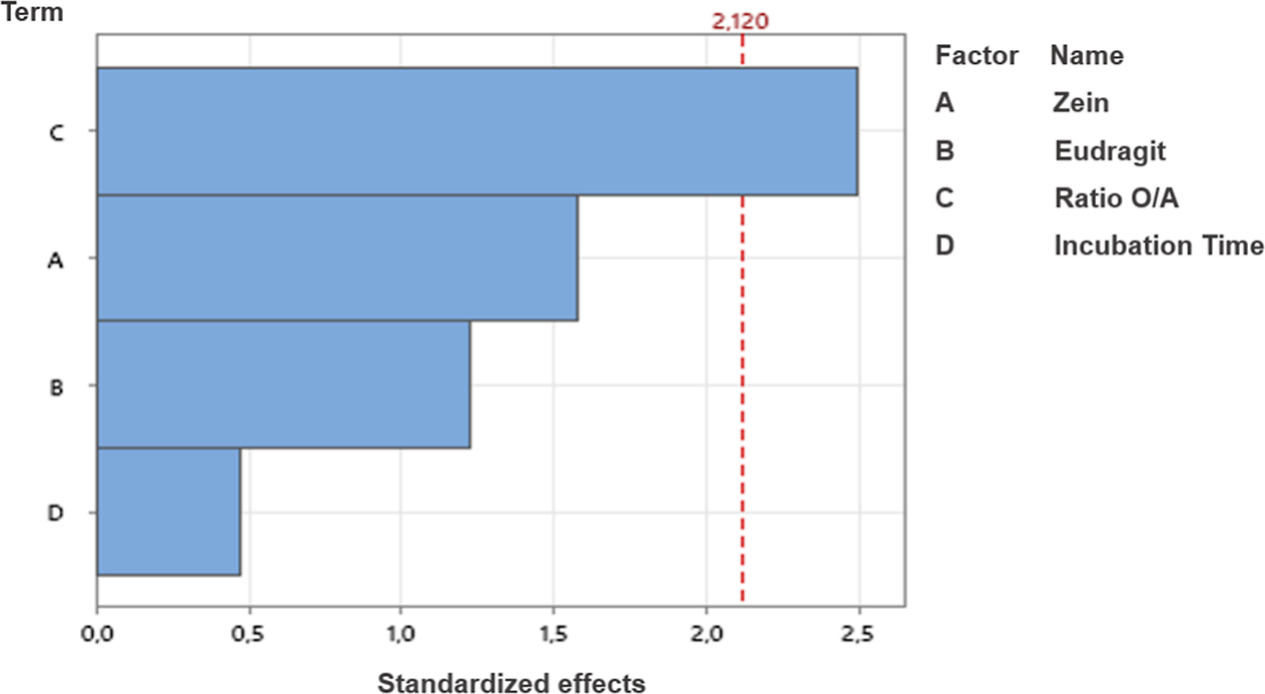
Pareto chart depicting the individual effects of zein, Eudragit, O/A ratio, and incubation time on GR.

As shown in Figure 2, the O/A ratio (Factor *C*) is the only variable demonstrating statistical significance (*p* < 0.05), as its bar exceeds the reference line. This highlights the critical role of the O/W ratio in influencing the GR. Consequently, adjusting the ratio of the organic phase to the aqueous phase is pivotal for optimizing the formulation process.

The main effects of each factor on the GR were further examined through a main effects plot (Figure 3).

**Figure 3:**
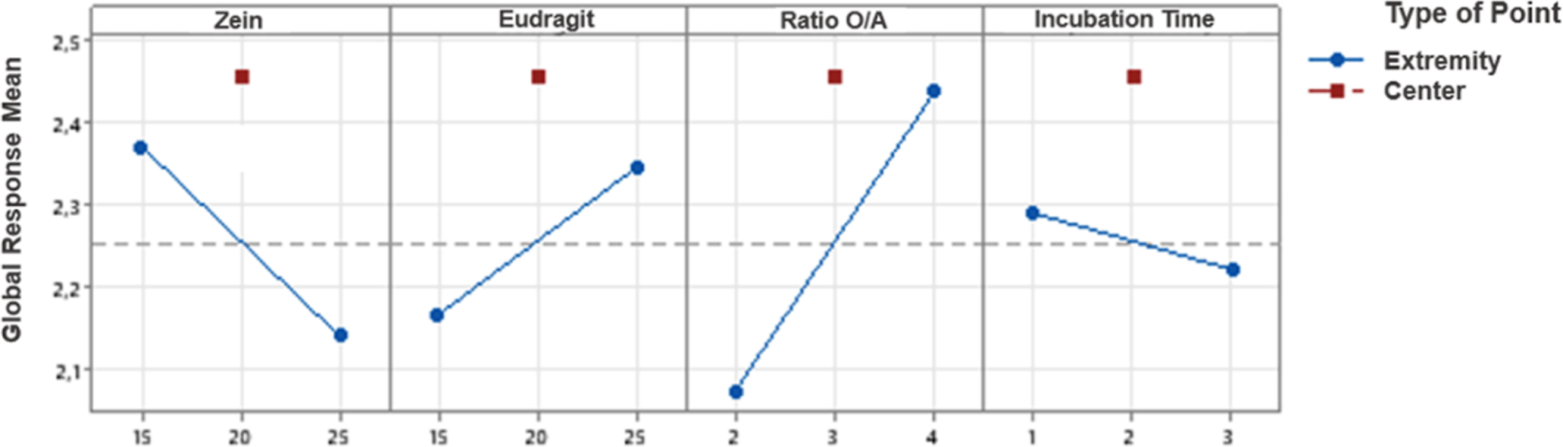
Main Effects Plot for GR. The blue dots indicate the conditions at the extremes of the levels tested for each factor, while the red dots represent the central conditions of the experimental design. The dotted line shows the global mean of the response, serving as a reference to compare the observed variations.

The main effects plot indicates zein concentration, Eudragit concentration, and the incubation time also significantly influence the GR (*p* < 0.05). Specifically, an increase in zein concentration results in a decrease in the GR, while an increase in Eudragit concentration leads to an increase in the GR. Incubation time shows a negative correlation with GR as well. However, the most substantial effect on GR is seen with O/A ratio, where an increase in the aqueous phase leads to a corresponding increase in the GR. These results suggest that careful modulation of these factors can significantly impact the overall success of the nanoparticle formulation process.

#### 3.1.2 Response Optimization

To identify the optimal conditions for preparing ZEU/INS NPS, a composite desirability function was used, aiming to minimize the GR. The target parameters for optimization were set as follows: EE: 40%, zeta potential: +30 mV, PDI: 0.3, mean size: 120 nm.

The optimization results (Table 3) indicate that the central point of the factors A (zein concentration), B (Eudragit concentration), C (O/A ratio), and D (incubation time) provides the best conditions for obtaining the desired nanoparticle characteristics. Desirability analysis, which ranges from 0 to 1, was used to evaluate the optimal set of responses, with 1 representing the ideal scenario. The composite desirability value obtained was close to 1, indicating that the predicted conditions closely align with the target values.

This analysis confirms that adjusting the formulation parameters, particularly maintaining the O/A ratio and balancing the concentrations of zein and Eudragit, allows for optimization of the ZEU/INS NPS. The results suggest that the central levels of these factors are sufficient to achieve high EE, suitable particle size, and stable zeta potential, all of which are critical for the successful intranasal delivery of insulin. The desirability function indicates that these conditions are optimal for the preparation of stable and effective insulin-loaded NPS, as evidenced by the high desirability score obtained.

### 3.2 Physicochemical Characterization of ZEU/INS NPS

For effective intranasal drug delivery, nanoparticle size plays a critical role in enhancing interaction with the nasal mucosa. NPS with smaller diameters are generally preferred, as they demonstrate better absorption across the nasal epithelium compared with larger particles. It is widely recommended that NPS designed for intranasal administration should not exceed an average diameter of 200 nm to ensure efficient uptake.^23,24^ In this study, the mean diameters of the ZEU/INS NPS were well within this recommended range, as shown in Table 4 and Figure 4, indicating their suitability for intranasal administration. The unimodal size distribution of these NPS further confirms their homogeneity and appropriateness for this route of delivery.

**Table 4:** Mean Size, PDI, and Zeta Potential of NPS (*n* = 3)^a^

formulation	mean size (nm)	PDI	zeta potential (mV)
ZEU NPS	125 ± 15^a^	0.23 ± 0.01^a^	+31.0 ± 1.5^a^
ZEU/INS NPS	133 ± 19^b^	0.28 ± 0.02^b^	+30.0 ± 1.7^a^

a ^a,b^Mean size, PDI, zeta potential ± standard deviation analyzed by column. Same letters indicate statistical equality, while different letters indicate statistical inequality (one-way ANOVA with Tukey post-test, *p* < 0.05).

**Figure 4:**
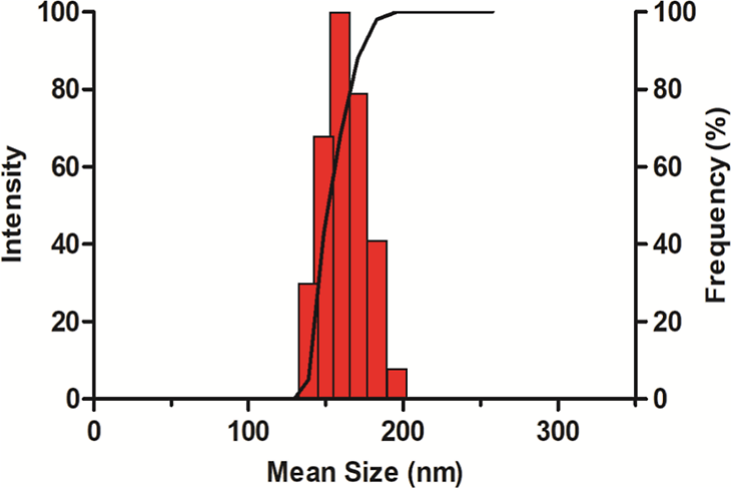
Size distribution of a representative ZEU/INS NPS sample.

The PDI values, which reflect the uniformity of nanoparticle size distribution, were significantly higher in the ZEU/INS NPS compared to those of the blank NPS (ZEU NPS). This increase in PDI can be attributed to the addition of insulin to the formulation, which introduces variability in nanoparticle size due to the insulin encapsulation process.^25^ However, despite this increase, the PDI values remained within an acceptable range, indicating that the formulation maintains a reasonably uniform particle size distribution.

Zeta potential measurements are crucial for understanding the stability of NPS in a suspension. The ZEU/INS NPS formulation exhibited a zeta potential of +30 mV, a result consistent with the presence of Eudragit, which is known to enhance the positive charge of the NPS. This occurs because zein, which has a neutral isoelectric point, contributes minimally to the overall surface charge, while the Eudragit component provides a stable positive charge.^26^ A zeta potential of ±30 mV is commonly regarded as a threshold for nanoparticle stability, where values beyond this range indicate sufficient electrostatic repulsion to prevent aggregation. In this case, a +30 mV zeta potential suggests that the ZEU/INS NPS possess good colloidal stability, reducing the likelihood of aggregation, coagulation, or flocculation.^27^ The positive surface charge of the NPS also enhances their interaction with the nasal mucosa. Mucin, the primary glycoprotein in mucus, carries a negative surface charge due to its isoelectric point (pI = 3). This negative charge facilitates strong electrostatic adhesion between mucin and the positively charged NPS, potentially prolonging the residence time of ZEU/INS NPS on the nasal mucosal surface.^28^ This prolonged contact is advantageous for intranasal delivery as it may enhance the bioavailability of insulin by increasing its absorption across the nasal epithelium.

The EE for insulin in the ZEU/INS NPS formulation was found to be 41.0% ± 0.7, highlighting a challenge often encountered when encapsulating highly hydrophilic molecules like insulin. Due to its strong affinity for the aqueous phase, insulin tends to partition out of the organic phase during nanoparticle formation, reducing the overall EE.^27,29^

#### 3.2.1 Scanning Electron Microscopy (SEM)

The morphological analysis of ZEU/INS NPS revealed an irregular particle profile (Figure 5), which is consistent with the observations of Sun et al.^23^ They reported that zein NPS tend to adopt irregular shapes when dispersed in ethanol, resulting in particles of various sizes. This variation in size distribution may be attributed to the dynamic nature of zein during the nanoparticle formation process, where ethanol influences the aggregation and fusion of the particles. A significant degree of nanoparticle aggregation was observed, which can be linked to the inherent amphiphilic nature of zein, as described by Li et al.^30^ The high density of hydroxyl groups on the surface of zein NPS promotes hydrophilic interactions, which leads to particle aggregation. These findings are consistent with other studies, such as those conducted by Qin et al.,^23^ Chang et al.,^24^ and Wang and Padua.^25^

**Figure 5:**
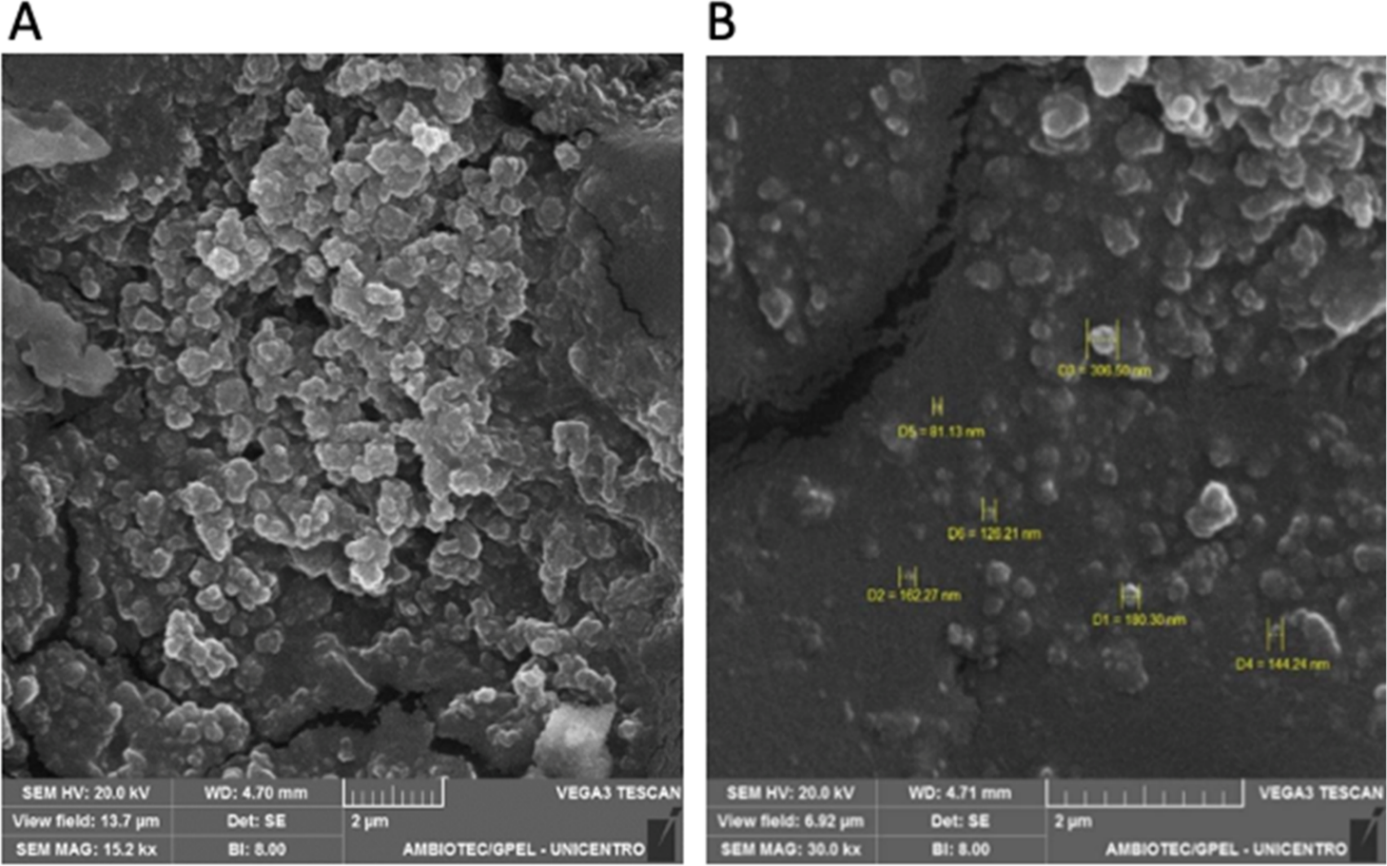
SEM micrographs of the prepared Zein/Eudragit composite NPS containing insulin at different magnifications: (A) 15,000×; (B) 30,000×.

The aggregation observed in this study is further explained by the fact that zein NPS are prone to aggregation when prepared without the inclusion of a nonionic surfactant. In the absence of a stabilizing agent, the hydrophobic regions of the zein molecules tend to interact, resulting in aggregation due to hydrophobic attraction between nonpolar groups on the particle surfaces.^26^ These results corroborate earlier reports by Bao et al.,^26^ where zein NPS exhibited elongated shapes, likely resulting from the fusion of multiple particles during the sample preparation process. Additionally, the SEM images showed that the NPS had a rough surface texture, supporting the findings of Reboredo et al.,^28^ who observed similar surface characteristics in zein-based NPS. This roughness is often attributed to the drying process or solvent evaporation, which causes surface irregularities and contributes to the formation of particle aggregates.

#### 3.2.2 Fourier Transform Infrared (FTIR) Spectroscopy

The FTIR spectra in Figure 6 represent the infrared absorption profiles of ZEU/INS NPS (A), ZEU NPS (B), insulin (C), zein (D), and Eudragit (E), and the physical mixture of zein and Eudragit (F). Each spectrum provides valuable information on the characteristic functional groups present in these formulations, helping us to understand possible chemical interactions between components in the NPS.

**Figure 6:**
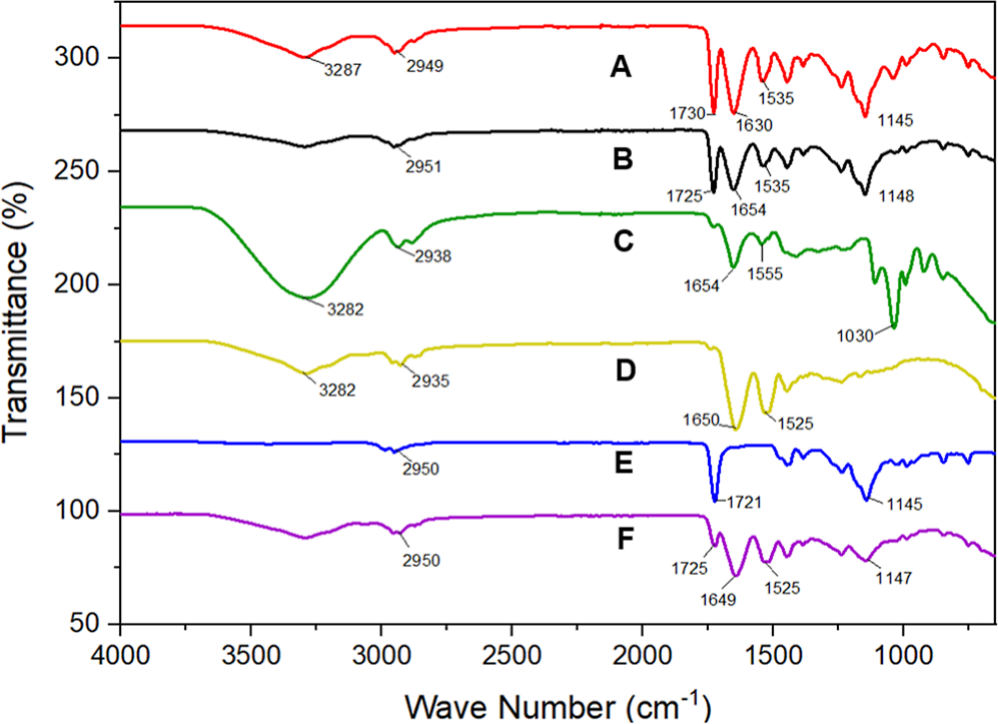
FTIR spectra of ZEU/INS NPS (A), ZEU NPS (B), insulin (C), zein (D), Eudragit (E), physical mixture (zein + Eudragit) (F).

In the FTIR spectrum of zein (Figure 6D), characteristic peaks emerge distinctly at specific intervals: at 2935 cm^–1^, the O–H stretching vibration is observed, reflecting the oscillations of methylene (CH_2_) and methyl (CH_3_) groups present in the side chains of amino acids in the zein molecule. The prominent C=O stretch at 1650 cm^–1^ highlights the presence of amide I-type peptide bonds in the protein structure. The 1525 cm^–1^ region is associated with the tyrosine band, linked to the aromatic ring vibration of tyrosine, whose side chain contains a phenol (−OH) group, and the Amide II band further confirms the proteinaceous nature of zein. Finally, the 3282 cm^–1^ range represents the O–H bond stretching of hydroxyl groups present in serine and threonine residues, which may also be present in the structure of insulin.^30,31^

The spectrum of the Eudragit (Figure 6E) shows different spectral intervals. At 1721 cm^–1^, the C=O stretch associated with ester groups present in the polymeric structure is highlighted. The 1145 cm^–1^ region identifies the C–O–C stretch, related to the ether groups present in the molecular configuration of Eudragit RS 100. Additionally, the 2950 cm^–1^ range shows the C–H stretching, similar to zein, reflecting the vibrations of methylene (CH_2_) and methyl (CH_3_) groups, but associated with groups present in this specific polymer.^16,32,33^

The spectrum of insulin (Figure 6C) obtained an O–H bond stretching of the hydroxyl groups present in serine and threonine residues in the 3282 cm^–1^ range. The 1654 cm^–1^ range indicates an amide I band (C=O bond), similar to that of zein, associated with peptide bonds. The amide II band (1555 cm^–1^) is also related to peptide bonds (N–H bending and C–N stretching). This band complements the information from amide I about the secondary structure of insulin. The 2938 cm^–1^ range reflects the C–H stretching band (vibrations of methylene (CH_2_) and methyl (CH_3_) groups in the amino acid side chains present in the structure of insulin). There was also a C–O stretching band at 1030 cm^–1^, which is often associated with the stretching of C–O bonds in ether or alcohol groups, such as glycerol that may be present in the insulin molecule.^34^

The spectrum of the physical mixture (zein + Eudragit) (Figure 6F) suggests a possible interaction between zein and Eudragit, with the shift of the Eudragit band from 1721 cm^–1^ to 1725 cm^–1^ (attributed to the C=O stretching of ester groups) and from 1145 cm^–1^ to 1147 cm^–1^ (attributed to C–O stretching). These small but distinct shifts suggest changes in the chemical environment of Eudragit’s functional groups, likely due to interactions with zein, such as hydrogen bonding or dipole–dipole interactions. These interactions may contribute to the stabilization of the nanoparticle matrix.

The ZEU/INS NPS spectrum (Figure 6A) combines the characteristic peaks of insulin, zein, and Eudragit, confirming the successful encapsulation of insulin within the nanoparticle matrix. The Amide I and II bands (1630 cm^–1^ and 1535 cm^–1^) confirm the presence of proteins (insulin and zein), while the C=O stretching at 1730 cm^–1^ and C–O–C stretching at 1145 cm^–1^ confirm the incorporation of Eudragit. The slight shifts in these peaks compared to the ZEU NPS (Figure 6B) and physical mixture (Figure 6F) suggest interactions between insulin and the nanoparticle components, likely due to the encapsulation process. In ZEU NPS, the Eudragit band shifted from 1721 cm^–1^ to 1725 cm^–1^, as in the physical mixture, while in ZEU/INS NPS, the band shifted to 1730 cm^–1^. This interaction can be attributed to hydrogen bonds, electrostatic interactions, and/or hydrophobic interactions, potentially indicating a significant interaction between zein and Eudragit.^35–37^ In ZEU/INS NPS and ZEU NPS, the band shifted from 1525 cm^–1^ to 1535 cm^–1^, which may be attributed to the C–C stretching vibration of the phenyl ring in tyrosine. Tyrosine is one of the amino acids that make up proteins and is known for its contribution to the structure and function of proteins.^31,38^ The shift of the 1650 cm^–1^ band to 1630 cm^–1^ in ZEU/INS NPS may be associated with electrostatic repulsion interactions between zein and insulin, as stated by Ji et al.^14^ This shift was not observed in ZEU NPS or the physical mixture, suggesting chemical interactions between zein and insulin in the ZEU/INS NPS.

Overall, the FTIR analysis confirms that insulin is successfully encapsulated within the ZEU/INS NPS and that the components of the formulation chemically interact to form a stable drug delivery system.

#### 3.2.3 Thermal Analysis

##### 3.2.3.1 Thermogravimetric Analysis (TG)

TG/DTG curves of zein (Figure 7C) show an initial weight loss (10%) at 76 °C, which may be attributed to the loss of adsorbed moisture due to its hygroscopic nature. The main decomposition event for zein occurs at 337 °C, indicating the breakdown of its protein structure.^39^

**Figure 7:**
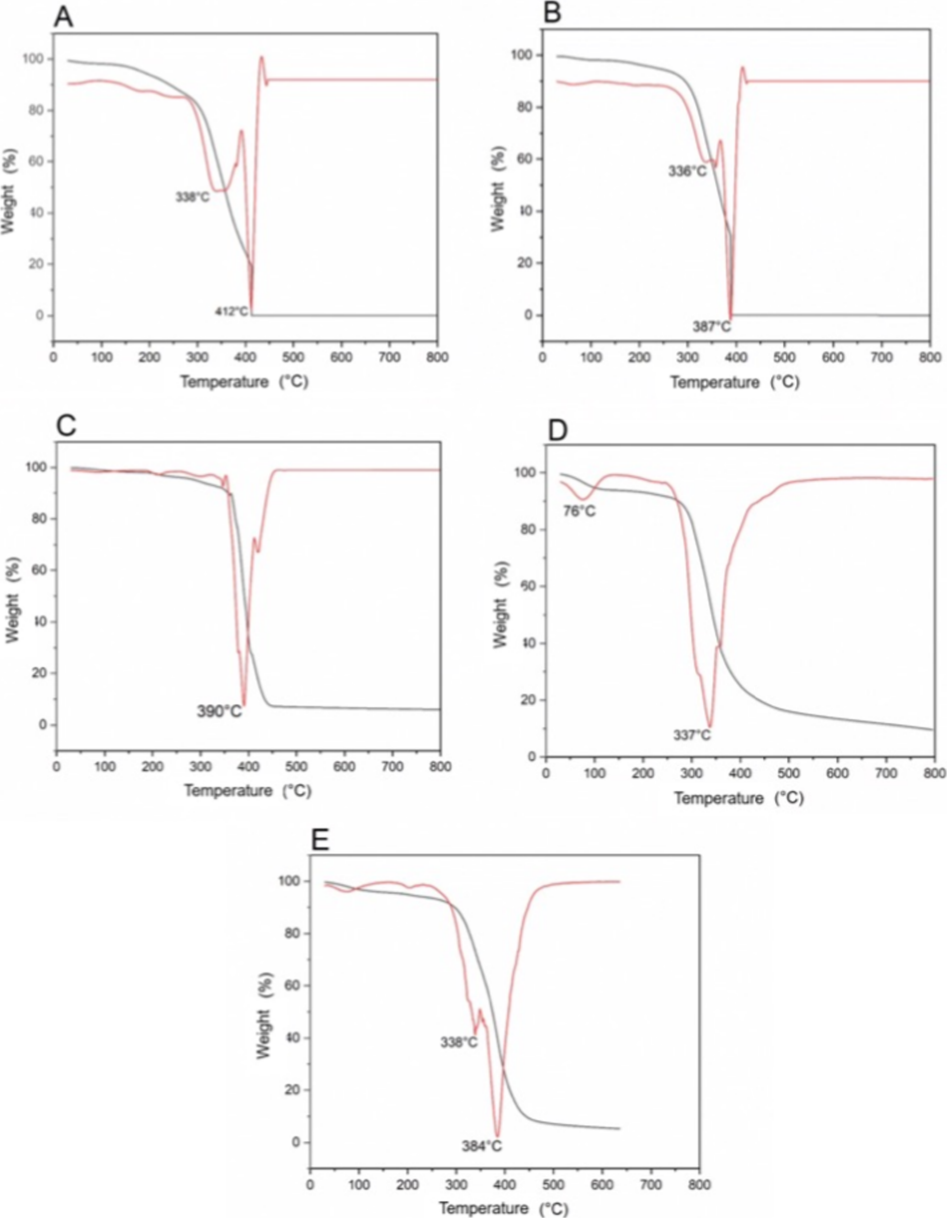
TG/DTG curves of ZEU/INS NPS (A), ZEU NPS (B), zein (C), Eudragit (D), physical mixture (zein + Eudragit) (E).

Eudragit alone exhibits excellent thermal stability, with its primary weight loss starting at 390 °C (Figure 7D). This higher decomposition temperature demonstrates the inherent stability of Eudragit, which is known for its use in drug delivery systems due to its ability to withstand high temperatures without significant degradation.

The TG/DTG curve of the physical mixture (Figure 7E) shows thermal behavior similar to the individual samples, with an initial weight loss (57%) at 338 °C, reflecting the decomposition of zein and at 384 °C for Eudragit.

The TG/DTG curve for ZEU/INS NPS shows the first major weight loss (52%) at around 338 °C, corresponding to the degradation of the zein. The lower thermal stability of pure zein compared to the ZEU/INS NPS and ZEU NPS formulations suggests that the interaction of zein with Eudragit in the NPS enhances its thermal stability. The second weight loss event occurs at 412 °C, indicating the thermal breakdown of residual components, zein, Eudragit, and potentially insulin. This finding is in line with the conclusions of Liu and Zhang,^40^ which indicates that the degradation of insulin occurs around 400 °C.

The TG curve for ZEU NPS shows thermal behavior similar to that of ZEU/INS NPS, with a primary weight loss event starting at 336 °C (related to zein degradation). The secondary weight loss occurs at a lower temperature (387 °C) compared to the insulin-loaded formulation, indicating that the absence of insulin slightly shifts the degradation process.

In conclusion, these results confirm that ZEU/INS NPS possess excellent thermal stability, making them suitable for applications requiring high-temperature processing or storage. The interaction between zein and Eudragit in the nanoparticle matrix enhances the thermal properties compared with their physical mixture or individual components.

##### 3.2.3.2 Differential Scanning Calorimetry (DSC)

DSC profiles shown in Figure 8 provide insight into the thermal behavior of ZEU/INS NPS and their individual components, including the blank NPS (ZEU NPS), zein, Eudragit, and a physical mixture of zein and Eudragit. By analyzing the onset of thermal transitions, peak temperatures, and enthalpy changes, we can infer key information about the thermal stability, drug–polymer interactions, and potential changes in the crystalline or amorphous nature of the NPS.

**Figure 8:**
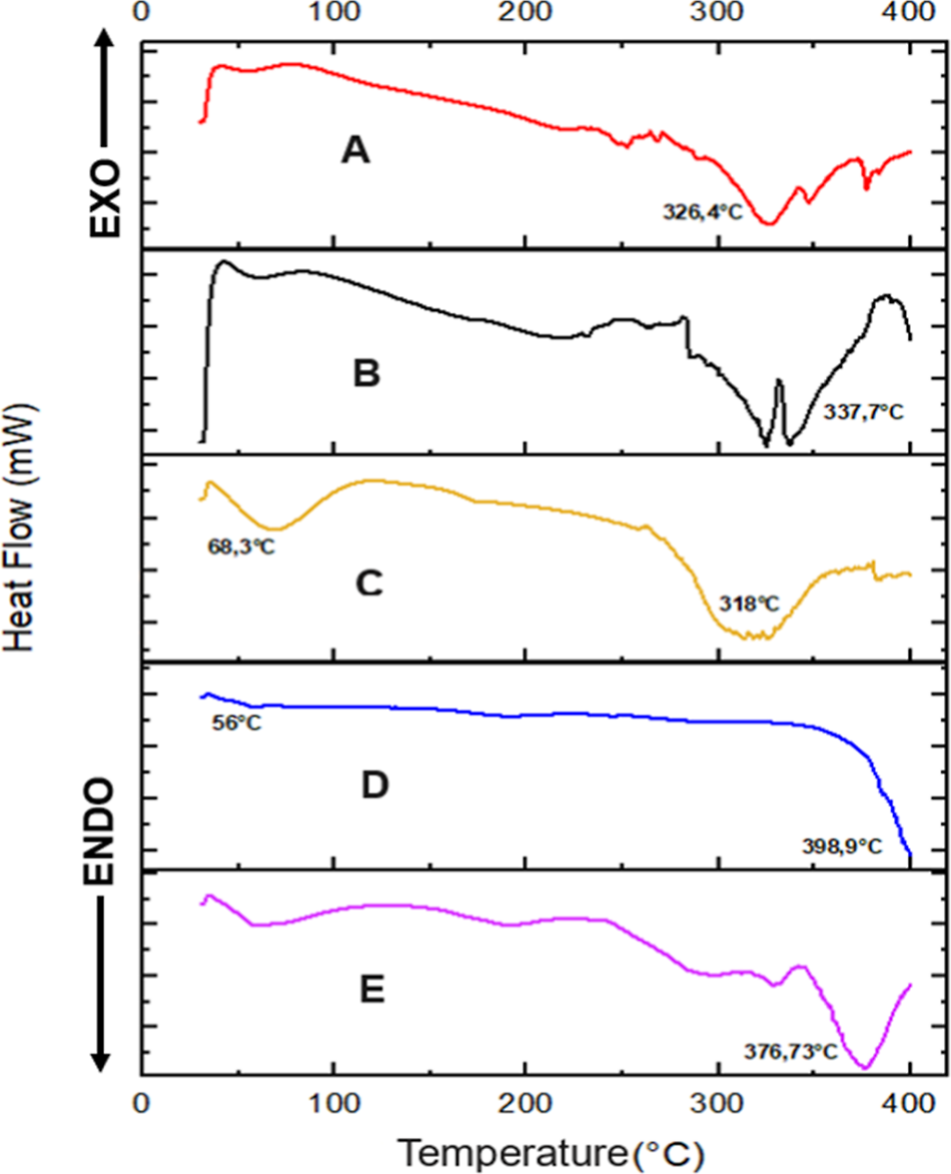
DSC curves of ZEU/INS NPS (A), ZEU NPS (B), zein (C), Eudragit (D), and physical mixture (zein + Eudragit) (E).

The DSC curve of pure Eudragit (Figure 8D) shows a sharp endothermic peak at 398.9 °C, indicating the thermal degradation of the polymer. Eudragit exhibits excellent thermal stability, with a high degradation temperature.^41^ This is an expected behavior for synthetic polymers such as Eudragit, which are often used for their stability in drug delivery systems. The glass transition (*T*_g_) in 56 °C was also observed.^42,43^

The DSC profile of zein (Figure 8C) shows an endothermic peak at approximately 68.3 °C, likely associated with the evaporation of water from the zein structure.^44^ The endothermic peak at 318 °C corresponds to the thermal degradation of zein, consistent with results from Shinde et al.^45^ and Elmizadeh et al.^32^

The physical mixture of zein and Eudragit (Figure 8E) displays a thermal interaction, resulting in thermal degradation of both compounds around 376.73 °C, similar to the study by Karthikeyan et al.^36^

The DSC curve for ZEU/INS NPS (Figure 8A) displays an endothermic peak at 326.4 °C, indicating the thermal degradation of the insulin-loaded nanoparticle system. This peak suggests that the nanoparticle formulation is thermally stable up to this temperature. This shift in degradation temperature implies strong interactions between insulin, zein, and Eudragit. The absence of sharp, distinct melting peaks for insulin indicates that the drug may be molecularly dispersed within the nanoparticle matrix in an amorphous or semicrystalline state, likely due to encapsulation.

The DSC profile of the ZEU NPS (Figure 8B) shows a thermal event at 337.7 °C, slightly higher than the insulin-loaded formulation. This suggests that the incorporation of insulin marginally lowers the degradation temperature, which is common when the drug interacts with the polymer matrix. The absence of additional melting transitions indicates that the blank NPS are predominantly amorphous with no crystalline polymer structures present.

The DSC analysis highlights important changes in the thermal behavior of ZEU/INS NPS compared with the individual components and their physical mixture. The increase in thermal stability observed in the ZEU/INS NPS and ZEU NPS samples, as evidenced by the higher degradation temperatures compared to those of zein, suggests significant interactions between the polymers (zein and Eudragit) and insulin. These interactions likely lead to a more stable, amorphous system, where the drug is molecularly dispersed within the matrix. The absence of distinct melting peaks for insulin in the ZEU/INS NPS formulation indicates that insulin is likely in an amorphous form, which could enhance its bioavailability. The high thermal stability of the formulation makes it suitable for drug delivery applications, where processing temperatures may be elevated.

In conclusion, the DSC profiles provide valuable insight into the stability and interactions within the ZEU/INS NPS, confirming that the encapsulation process enhances the thermal properties of zein and Eudragit and results in a stable, amorphous insulin-loaded nanoparticle formulation.

### 3.3 Evaluation of Stability

The stability study of ZEU/INS NPS was conducted over 63 days under two different storage conditions: refrigeration and ambient temperature. The parameters evaluated include the mean particle size, PDI, zeta potential, and insulin content, as presented in Figure 9.

**Figure 9:**
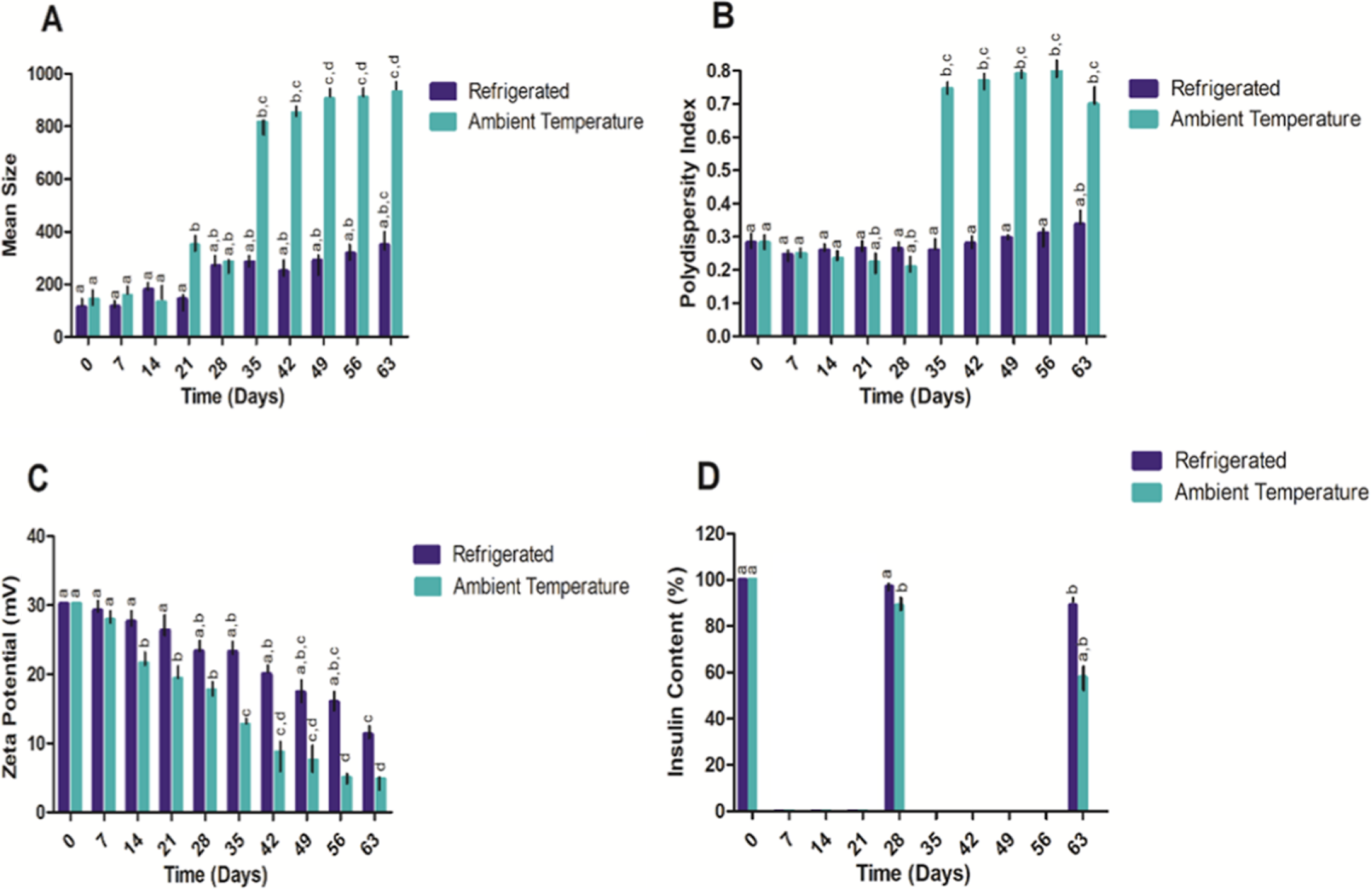
ZEU/INS NPS under different storage conditions (*n* = 3). Mean diameter (A), PDI (B), zeta potential (C), drug content (D). ^a,b,c,d^Same letters indicate statistical equality and different letters indicate statistical inequality (one-way ANOVA with Tukey’s post-test and α < 0.05).

Initially, the mean size of the NPS remained stable at around 160 nm with no significant differences between the refrigerated and ambient temperature conditions during the first 28 days (Figure 9A). However, from day 35 onward, a marked increase in particle size was observed for samples stored at ambient temperature, reaching over 800 nm by day 63. NPS stored under refrigeration showed a slight increase in size after 28 days. These results suggest that the higher temperature at ambient conditions may accelerate nanoparticle aggregation, possibly due to increased kinetic energy, leading to instability over time. Refrigeration, on the other hand, appears to slow down this process, preserving the nanoparticle size more effectively.

The PDI values (Figure 9B), which indicate the homogeneity of the nanoparticle population, follow a trend similar to the particle size. At ambient temperature, there was a gradual increase in PDI from day 28 onward, peaking at around 0.7 by day 63, reflecting a higher degree of particle size heterogeneity. This suggests that nanoparticle aggregation leads to a more diverse particle population in terms of size, especially in the samples stored at ambient temperature. In contrast, the refrigerated samples showed minimal changes in PDI, maintaining values around 0.3, indicative of a more homogeneous particle distribution. This further supports the observation that refrigeration better preserves the physical stability of the NPS.

The zeta potential, a measure of the surface charge and colloidal stability of NPS, also displayed temperature-dependent trends (Figure 9C). Initially, both conditions exhibited a zeta potential of approximately +30 mV, indicative of stable NPS with good electrostatic repulsion to prevent aggregation. After 14 days, the zeta potential of the ambient temperature samples gradually decreased, dropping below +15 mV by day 63, indicating reduced electrostatic stability and a greater likelihood of aggregation. In contrast, the refrigerated samples maintained a zeta potential close to +25 mV until 42 days, suggesting that storage at lower temperatures helps retain the NPS’ surface charge and stability.

Insulin content (Figure 9D) remained relatively stable for the first 28 days in both storage conditions with an EE close to 100%. However, significant differences were observed from day 35 onward. The samples stored at ambient temperature exhibited a sharp decline in insulin content, dropping to approximately 40% by day 63. In contrast, the refrigerated samples retained around 80% of their insulin content at the end of the study period. This indicates that higher storage temperatures may accelerate the degradation or release of encapsulated insulin, whereas refrigeration significantly slows down these processes, preserving the drug’s stability within the NPS.

The results of this stability study demonstrate the significant impact of storage conditions on the physicochemical stability of ZEU/INS NPS. NPS stored at ambient temperature showed substantial increases in particle size and PDI, along with a decrease in zeta potential and insulin content, all of which are indicative of decreased stability. These changes suggest that ambient temperature leads to nanoparticle aggregation, loss of colloidal stability, and degradation of the encapsulated drug. In contrast, NPS stored under refrigeration showed much better stability, with only slight increases in size and PDI, and minimal changes in the zeta potential and insulin content. These findings suggest that refrigeration is crucial for maintaining the physical and chemical stability of ZEU/INS NPS, likely by reducing the kinetic energy of the system, slowing aggregation, and preventing insulin degradation.

### 3.4 In Vitro Release Study

The in vitro release profile of insulin from ZEU/INS NPS was evaluated using simulated nasal fluid with a pH of 6.5 at 37 °C. The results of the insulin release assay are presented in Figure 10.

**Figure 10:**
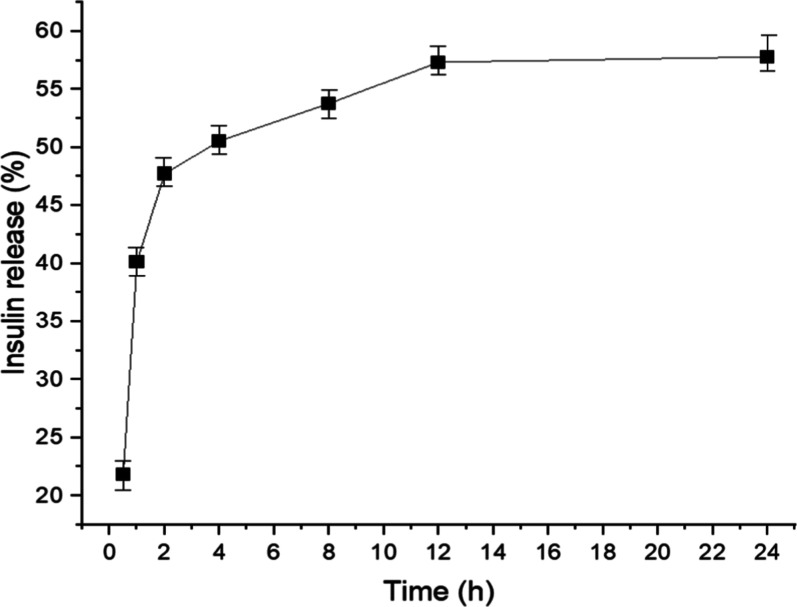
In vitro insulin release from ZEU/INS NPS in simulated nasal fluid (pH = 6.5) over 24 h (*n* = 3).

The release profile demonstrates a biphasic release pattern, consisting initially of a burst effect, followed by a slower subsequent release. During the first 4 h, there is a rapid release of insulin, with approximately 45% of the total insulin being released. This initial burst may be attributed to insulin present on or near the surface of the NPS, which is more readily available for release into the medium. Surface-associated insulin is often released quickly due to the diffusion process and immediate interaction with the receptor medium (simulated nasal fluid). The rapid initial release is common in nanoparticle systems, especially when a portion of the drug is weakly bound to the nanoparticle surface. In practical applications, this burst can be beneficial for quickly achieving therapeutic concentrations of the drug.

After the initial burst, the release rate slows down, with an additional 10% of insulin being released over the next 8 h. This slower, sustained release phase can be attributed to the gradual diffusion of insulin encapsulated within the nanoparticle core. This phase is crucial for ensuring prolonged therapeutic effects in intranasal drug delivery, where the drug is released over an extended period, improving bioavailability, and reducing the need for frequent administration.

The release curve plateaus after approximately 12 h, with the cumulative insulin release reaching ∼55–60%. The plateau suggests that a significant proportion of insulin remains encapsulated or is bound within the core of the NPS, which may be released slowly or not at all. This behavior is typical of polymer-based NPS, where complete release is often not achieved under in vitro conditions. However, the release rate may differ in vivo due to enzymatic degradation and other biological factors.

Overall, the ZEU/INS NPS formulation exhibits a controlled release profile with an initial burst followed by a more gradual and sustained release of insulin. This release behavior is promising for intranasal delivery, where a combination of rapid onset and prolonged effects can improve therapeutic outcomes in diseases such as diabetes. Further optimization of the nanoparticle formulation could focus on fine-tuning the balance between burst and sustained release to better suit specific clinical needs.

To analyze the release kinetics of insulin from ZEU/INS NPS, various mathematical models were applied to the in vitro release data using KinetDS software. The goal of the analysis was to determine the model that best describes the release mechanism, based on the correlation coefficient (*r*) values, with higher r values indicating a better fit. The mathematical models used and their respective “*r*” values are presented in Table 5.

**Table 5:** Kinetic Analysis of Insulin Release from ZEU/INS NPS in Simulated Nasal Fluid (pH = 6.5)^a^

model	*r*
zero-order	0.7798
first-order	0.5876
second-order	0.3919
third-order	0.2602
Higuchi	0.5929
Weibull	0.9608
Hickson-Crowell	0.6565
Baker-Lonsdale	0.9402
Korsmeyer-Peppas	0.9352

a Source: the author, via KinetDS (2024).

The Weibull model provided the highest correlation coefficient (*r* = 0.9608), suggesting that this model best fits the release kinetics of insulin from the ZEU/INS NPS. The Weibull model is often used to describe the time-dependent release of drugs from various delivery systems including polymers and NPS. This suggests a complex release mechanism that may involve the dissolution of insulin, the diffusion of insulin through the nanoparticle matrix, and the structural characteristics of the NPS themselves.^46,47^

The overall results indicate ZEU/INS NPS is potentially useful for sustained and controlled drug delivery applications.

### 3.5 In Vitro Mucoadhesion Study

The goal of this assay was to evaluate the mucoadhesive properties of ZEU/INS NPS in the presence of various concentrations of mucin type II after 30 min of incubation. Results are expressed in Table 6.

**Table 6:** Mean Size, PDI, and Zeta Potential of ZEU/INS NPS before and after Contact with Different Concentrations of Mucin

Mucin (μg/mL)	mean size ± SD (nm)	PDI ± SD	zeta potential ± SD (mV)
0	160 ± 24	0.30 ± 0.01	+29.3 ± 0.6
100	2564 ± 39	0.52 ± 0.05	–20.5 ± 1.2
250	3394 ± 17	0.61 ± 0.05	–15.7 ± 1.6
500	3431 ± 75	0.53 ± 0.04	–13.2 ± 0.9

Before contact with mucin, the NPS exhibited an average size of 160 nm, which is within the optimal size range for intranasal drug delivery. After contact with mucin, there is an increase in the particle size, indicating significant mucoadhesion. Also, a concentration-dependent increase in particle size is observed, which suggests that more mucin is available to interact with the NPS, forming larger mucin-nanoparticle aggregates. The PDI increased significantly with higher concentrations of mucin, reflecting a greater size variability due to the formation of aggregates. Before contact with mucin, the NPS exhibited a positive zeta potential of +29.3 mV, indicating good colloidal stability due to electrostatic repulsion. However, a substantial shift in zeta potential to the negative range was observed after interaction with mucin, which carries a negative charge. This shift indicates strong electrostatic interactions between the positively charged NPS and the negatively charged mucin. The decrease in zeta potential suggests that mucin adsorption onto the nanoparticle surface is progressively reducing the net positive charge of the NPS.^16^ These findings confirm that the NPS interact effectively with mucin, forming stable mucoadhesive complexes.

The concentration-dependent aggregation observed in this study suggests that ZEU/INS NPS is well-suited for intranasal drug delivery, where enhanced mucoadhesion can significantly improve drug retention and absorption at the nasal mucosa. Mucoadhesion plays a pivotal role in nasal drug delivery due to the nasal cavity’s protective mechanisms, including cilia and mucus, which act as the first line of defense against pathogens and particles. In the respiratory region, ciliary movement and mucus production contribute to mucociliary clearance, a critical defense process.^48^ For effective intranasal administration, drug formulations must be designed to overcome the challenges posed by mucociliary clearance, ensuring that they remain on the nasal mucosa long enough to be absorbed, permeated, and transported to the intended site of action.^17^ The ability of NPS to adhere to mucus is a key characteristic that enhances drug retention and absorption, prolonging their residence time on the nasal surface and, consequently, improving therapeutic efficacy.^49^

The combined results of this study suggest that the ZEU/INS NPS holds significant potential for intranasal insulin delivery. The optimized formulations demonstrated desirable physicochemical properties, including an appropriate particle size for crossing the nasal epithelium, a positive surface charge conducive to mucoadhesion, a high EE, and controlled release profiles. These attributes collectively align with the critical requirements for effective intranasal drug delivery systems. While these findings highlight the promise of this nanoparticle system, it is important to emphasize that further in-depth studies, including in vivo evaluations and long-term safety assessments, are necessary to confirm their efficacy and therapeutic potential.

## 4 Conclusion

This study successfully developed and characterized Zein/Eudragit composite NPS (ZEU/INS NPS) for the intranasal delivery of insulin, demonstrating their potential as an alternative to conventional parenteral administration for the treatment of T1DM. Through the application of a 2^4^ factorial design, we optimized the nanoparticle formulation process, resulting in a system with desirable physicochemical properties, including a particle size below 200 nm, a PDI under 0.3, a zeta potential of approximately +30 mV, and an EE of 42%. The in vitro release studies revealed a sustained insulin release profile, with 60% of the insulin released over 24 h, and the in vitro mucoadhesion assay confirmed the ability of the NPS to adhere to the nasal mucosa, promoting prolonged drug retention. Furthermore, the NPS exhibited good thermal stability and remained physically stable for up to 60 days under refrigerated conditions. Overall, these findings suggest that ZEU/INS NPS are a promising platform for intranasal insulin delivery, offering a patient-friendly, noninvasive alternative to injections. Further in vivo studies and clinical evaluations will be necessary to fully assess their therapeutic potential and optimize the formulation for long-term use in diabetic management. Nonetheless, this research represents a significant step toward the development of more effective and accessible insulin delivery systems for patients with Diabetes.
